# *Plasmodium* Reproduction, Cell Size, and Transcription: How to Cope With Increasing DNA Content?

**DOI:** 10.3389/fcimb.2021.660679

**Published:** 2021-04-09

**Authors:** Marta Machado, Salome Steinke, Markus Ganter

**Affiliations:** ^1^ Centre for Infectious Diseases, Parasitology, Heidelberg University Hospital, Heidelberg, Germany; ^2^ Graduate Program in Areas of Basic and Applied Biology, Instituto de Ciências Biomédicas Abel Salazar, Universidade do Porto, Porto, Portugal

**Keywords:** malaria, *Plasmodium*, polyploidy, DNA content, gene dosage, gene expression, transcription regulation

## Abstract

*Plasmodium*, the unicellular parasite that causes malaria, evolved a highly unusual mode of reproduction. During its complex life cycle, invasive or transmissive stages alternate with proliferating stages, where a single parasite can produce tens of thousands of progeny. In the clinically relevant blood stage of infection, the parasite replicates its genome up to thirty times and forms a multinucleated cell before daughter cells are assembled. Thus, within a single cell cycle, *Plasmodium* develops from a haploid to a polypoid cell, harboring multiple copies of its genome. Polyploidy creates several biological challenges, such as imbalances in genome output, and cells can respond to this by changing their size and/or alter the production of RNA species and protein to achieve expression homeostasis. However, the effects and possible adaptations of *Plasmodium* to the massively increasing DNA content are unknown. Here, we revisit and embed current *Plasmodium* literature in the context of polyploidy and propose potential mechanisms of the parasite to cope with the increasing gene dosage.

## Introduction

Malaria is caused by unicellular eukaryotic parasites of the genus *Plasmodium*. Several species of this evolutionary very distinct genus cause malaria in humans, with *Plasmodium falciparum* (*P. falciparum*) being the most virulent one. *Plasmodium* spp. display a complex life cycle, alternating between a mosquito and a vertebrate host. Observing the parasite population size throughout its life cycle reveals two striking numerical bottlenecks, which occur each time the parasite changes its host (Cowman et al., 2016; Matthews et al., 2018). To compensate for these losses the parasite proliferates massively. After transmission to the mosquito, one cell cycle produces hundreds to thousands of daughter cells in a stage called oocyst. After transmission to the human host, *P. falciparum* proliferates first in hepatocytes. Here, one cell cycle of the parasite can give rise to tens of thousands of daughter cells, which leave the liver and infect red blood cells, where they continue to proliferate. This additional proliferation establishes parasite densities in peripheral blood that are sufficiently high to ensure transmission during an ensuing mosquito blood meal. All three described parasite proliferation events result in the formation of a polyploid and multinucleated cell before daughter cells are assembled during a relatively synchronous mass cytokinesis event (Prudêncio et al., 2006; Burda et al., 2017; Spreng et al., 2019; Rudlaff et al., 2020; Simon et al., this issue). Thus, at a given time, the parasite cytoplasm can harbor one to dozens or even thousands of genome copies.

**Figure 1:**
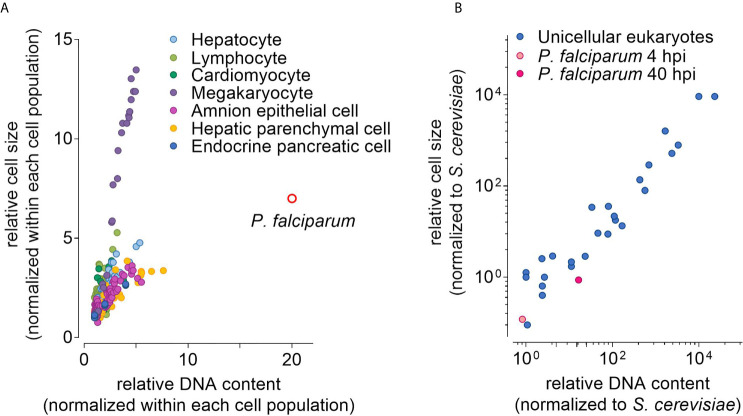
Distribution of nuclear DNA content and cell volume in eukaryotic cells. **(A)** Relative DNA content and cell size of *P. falciparum* (40 hours post invasion, hpi, of an erythrocyte) and human cells with varying ploidy based on Gillooly et al., 2015. Each point represents the fold increase in cell size and DNA content relative to the smallest cell with the lowest DNA content of each population. **(B)** Relative DNA content and cell size of 25 species of unicellular eukaryotes in G1 phase based on Table 2 from Shuter et al., 1983. Here, the relative cell size and DNA content was normalized to the *Saccharomyces cerevisiae*. Note: Values for *P. falciparum* were derived as follows: estimated DNA content before DNA replication in femtogram reported by Mangold et al., 2005; ploidy level based on Ganter et al., 2017 and Simon et al., this issue; cell volume estimated from Waldecker et al., 2017.

Other examples of cells (and organisms) with a ‘higher-than-usual’ DNA content can be found in many eukaryotes and on all levels of biological organization (Edgar et al., 2014; Fox et al., 2020). The cells of many plants, domesticated or not, are polyploid. In mammals, polyploid cells are found for example in the liver or in the bone marrow (Gillooly et al., 2015) (**Figure 1A**). Polyploidy increases the size of the cell and can allow for higher genomic output, i.e., RNA species and protein (Osborn et al., 2003; Comai, 2005; Otto, 2007; Parisod et al., 2010; Marshall et al., 2012; Edgar et al., 2014; Frawley and Orr-Weaver, 2015; Schoenfelder and Fox, 2015; Fox et al., 2020). For example, the silk gland cells of the silk worm *Bombyx mori* increase their genomic DNA content up to 4 x10^5^ times during larval development, enhancing the secretion of macromolecules (Gage, 1974).

When additional genomic output is not needed, an increased gene dosage can be detrimental, likely as a result of disrupted gene expression homeostasis (Sheltzer and Amon, 2011; Birchler and Veitia, 2012). This imbalance can have severe adverse effects, which can be seen in chromosomal disorders like trisomy. Here, the additional copy of a single chromosome leads to an imbalance in protein concentration, resulting in frequent miscarriages (Herbert et al., 2015). Sex chromosomes also cause an imbalance and compensatory mechanisms have evolved (Wutz and Gribnau, 2007; Lucchesi and Kuroda, 2015; Disteche, 2016). For example, in many female mammals compensation is achieved by the inactivation of one of the two X-chromosomes (Wutz and Gribnau, 2007). Conversely, in male *Drosophila* cells, transcription from the single X-chromosome is increased two-fold (Lucchesi and Kuroda, 2015).

However, our understanding of the impact of increasing DNA content for *Plasmodium* is very rudimentary and not discussed in the literature. Yet, investigating the effects of increasing DNA content, and thus gene dosage, is key to our understanding of other aspects of parasite biology. Hence, in this *Perspective* we discuss potential strategies of *Plasmodium* to cope with polyploidy and highlight some gaps in our knowledge of *Plasmodium* biology.

## Increased DNA Content and Cell Size

In many cells, polyploidy is associated with an increased cell size. While the causal relationship between the two remains unclear (Sugimoto-Shirasu and Roberts, 2003; Wood and Nurse, 2015), a positive correlation between DNA content and cell size can be observed in bacteria, plants and mammals (Jovtchev et al., 2006; Fomina-Yadlin et al., 2014). Strikingly, this can also be seen in cells of the same type, for example human megakaryocytes vary dramatically in their ploidy level with megakaryocytes of higher ploidy being substantially larger than megakaryocytes of lower ploidy (Gillooly et al., 2015). Thus, increasing the cell size may be a strategy for *P. falciparum* to cope with the increasing DNA content.

Data to test this hypothesis are scarce, especially for the proliferating parasite stages in the mosquito and the liver. For blood stage parasites, several studies report that the DNA content (or the number of daughter cells as proxy) increases 20-fold on average within one proliferative cycle and individual cells with an up to 30-fold increase can be found occasionally (Ganter et al., 2017; Simon et al., in this issue). But, to our knowledge, only Waldecker et al. quantified the volume occupied by *P. falciparum* inside the erythrocyte over time (Waldecker et al., 2017). They found that blood stage parasites grow approximately 3-fold during the so-called ring and trophozoite stage, and approximately 7-fold during the complete proliferative cycle. Whether this 7-fold increase in cell size is sufficient to cope with the up to 30-fold increase in DNA content is difficult to gauge. Human cells of the same type but with different ploidy levels resemble somewhat the situation in the parasite, in terms of the change in DNA content over time (in contrast to polyploid species, where the level of polyploidy is stable over time). Plotting the relative cell size versus relative DNA content, *P*. *falciparum* clearly segregates from human cells, as the parasite displays a comparatively small increase in size in relation to the DNA content (Gillooly et al., 2015) (**Figure 1A**). This would argue against increasing cell size as major coping mechanism. Nevertheless, the sizes and DNA contents of the developing *P. falciparum* blood-stage parasite still fall within the range of size vs. DNA content of other unicellular eukaryotes (Shuter et al., 1983) (**Figure 1B**). Whether this interspecies comparison is valid remains questionable, since only the pre-DNA replication values were reported for the other unicellular eukaryotes. Another major caveat is the comparison of a relatively stable situation with dynamic events, as both size and DNA content change quickly within one proliferative cycle of the parasite (Ganter et al., 2017; Waldecker et al., 2017). Thus, it remains unclear, whether increasing cell size plays a role for coping with increasing DNA content in the blood stage of infection. High- and super resolution microscopy may allow for the parallel quantification of cell size and DNA content of replicating parasites in the blood, mosquito, and liver stage. These analyses will provide valuable insights to better understand the interplay between cell size and DNA content.

## Increased DNA Content and Expression Homeostasis

**Figure 2:**
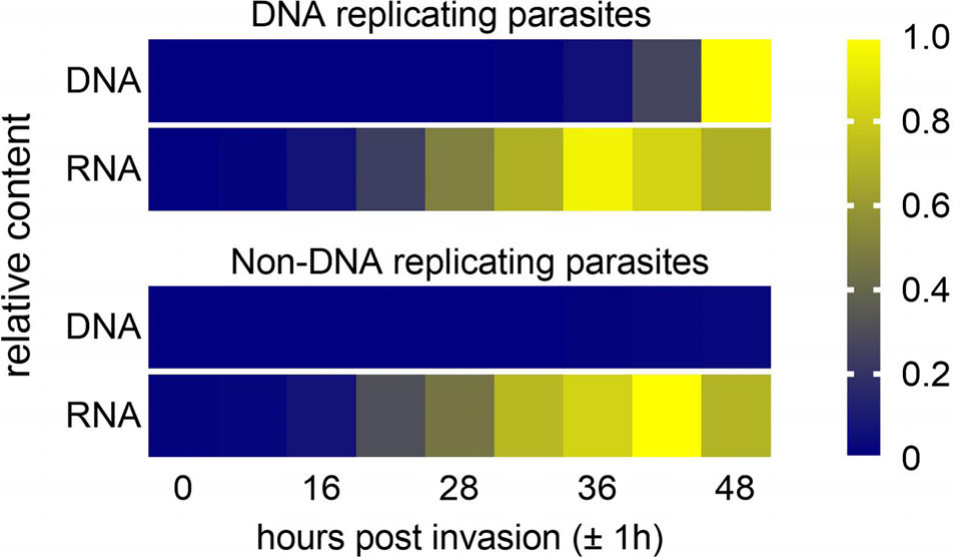
Estimation of total DNA and total RNA content during a proliferative cycle of *P. falciparum* blood stages. Synchronized cultures of DNA replicating (PfCRK4-DD on Shield-1) and non-DNA replicating (PfCRK4-DD off Shield-1; Ganter et al., 2017) parasites were treated with or without RNase. Subsequently, parasites were stained with SYBR green I, which labels both DNA and RNA (Theron et al., 2010; Ganter et al., 2017) and analyzed by flow cytometry. Total DNA and total RNA contents were estimated by calculating the difference in signal intensity with and without RNase treatment. Total DNA and total RNA contents were normalized by Min-Max scaling using the minimum and maximum values for DNA and RNA regardless of whether parasites replicate DNA or not.

During S-phase of the cell cycle and before cytokinesis, a similar dynamic change of DNA content also occurs: While cells grow continuously, their DNA content doubles abruptly (Bar-Ziv et al., 2016). For bacteria it was shown that the expression of a given gene increases shortly after it is replicated (Chandler and Pritchard, 1975; Schmid and Roth, 1987). Eukaryotic transcription, however, is buffered against increasing gene dosage during S-phase (Killander and Zetterberg, 1965; Elliott and McLaughlin, 1978; Bar-Ziv et al., 2016). It was shown for *Saccharomyces cerevisiae* that transcription from recently synthetized DNA is reduced, which depends on acetylation of a histone H3 lysine residue at position 56 (Voichek et al., 2016). Thus, we asked whether altering the genome output could also be a mechanism employed by *P. falciparum* to cope with the increasing DNA content. This hypothesis would predict little or no correlation between DNA and total RNA content of the cell. This notion is supported by a study from the mid 1980’s, which found the rate of total RNA synthesis to peak at approximately 38 hours post invasion (hpi), while the rate of DNA replication peaked at approximately 45 hpi (De Rojas and Wasserman, 1985). Employing nuclear run-on assays Sims et al., 2009 reported highest transcriptional activity in parasites with a 10-fold increased DNA content, but a linear correlation between the number of nuclei per cell and RNA polymerase II-dependent transcriptional activity was not found (Sims et al., 2009). This may indicate a mechanism that balances DNA content and the transcription of messenger RNA (mRNA). To provide more insights into the relationship of total DNA and total RNA content over time during parasite development in the blood stage, we measured the DNA and, indirectly, the total RNA content by flow cytometry. This estimation found that the total RNA content peaked approximately 12 hours before the DNA content reached a maximum (**Figure 2**, top panel). In addition, similar RNA contents were also reached in parasites where DNA replication was genetically blocked (**Figure 2**, bottom panel). Together this suggest limited or no scaling of total RNA content and total DNA content and strongly supports the existence of a mechanism that buffers genome output against increasing DNA content in *P. falciparum*. In such a scenario, the parasite could use shared pools of nucleotide precursors more efficiently. Yet, to be able to draw firm conclusions, more data are needed on the correlation of total RNA and total DNA content over time. In addition, quantifying the relative amount of the different RNA species in relation to the DNA content will not only inform on the relative contribution to the total RNA content but may also shed light on the regulatory mechanism that drive the expression of the different RNA species.

Two other observations support the hypothesis of a reduced genome output in parasites with an increased DNA content. Firstly, the distribution and number of nuclear pore complexes (NPCs) is highly dynamic during one proliferative cycle. The number of NPCs per nucleus peaks in late trophozoites (1 to 2 genome copies) and then decrease gradually as nuclei divide (Weiner et al., 2011; Dahan-Pasternak et al., 2013; Guizetti et al., 2013; Hollin and Le Roch, 2020). It is also at the late trophozoite stage when NPCs show a redistribution from a polarized to a more dispersed localization across the nuclei (Weiner et al., 2011). Interestingly, these phenomena coincide with the peak in total RNA content (**Figure 2**). Thus it is reasonable to assume that gene gating also occurs in *Plasmodium* (Blobel, 1985; Cabal et al., 2006; Mendjan et al., 2006; Weiner et al., 2011; Dahan-Pasternak et al., 2013; Raices and D’Angelo, 2017) and that the NPCs are potentially involved in the regulation of gene expression.

Secondly, transcription in *P. falciparum* was described as not responsive to environmental perturbations (Gunasekera et al., 2007; Ganesan et al., 2008; Young et al., 2008). For cells with a changing DNA content, being not responsive may be beneficial as it intuitively appears difficult to equally integrate an external stimulus in a cell with one or multiple copies of the genome. Nevertheless, the significance of these observations in the context of polyploidy and genome output remains unclear.

Another observation contributes to our picture of transcription in polyploid *P. falciparum* during the blood stage. Labeling of nascent RNA with 5-bromouridine 5′-triphosphate (BrUTP) reported an uneven staining among the different nuclei of the parasite, suggesting differential transcriptional activity (Moraes et al., 2013). This indicates that there may be unexplored means of transcriptional regulation in multinucleated stages. To further investigate potential qualitative and quantitative transcriptional differences between nuclei of a given cell, more experiments are needed. For example, investigating all three *Plasmodium* RNA polymerases (I, II and III) and assessing their spatiotemporal localization dynamics in multinucleated cells will be informative (Zhao et al., 2016). Moreover, single-molecule fluorescence *in situ* hybridization (smFISH) of different RNA transcripts (e.g., ‘housekeeping’ genes and stage specific genes) can provide high-resolution snapshots of the transcription and localization of different RNAs at defined time points (Kramer et al., 2020). Integrating such data with RNA species quantification in blood parasites and also liver and mosquito stages, will shed light on the potential mechanisms that are in play to buffer against the increasing DNA content.

## mRNA Transcription as Proxy for Genome Output

The apparent independence of total RNA content and total DNA content over time could be achieved in different ways, which individually or in concert may help to maintain gene expression homeostasis. The total RNA output could be altered by activating only a subset of genomes or all genomes transcribe RNA, but each produces less. How exactly the parasite responds to the increasing DNA content is unknown but cues may be found in the literature on *Plasmodium* mRNA transcription. To gain insight, we need to compare temporal information on mRNA transcription and DNA content, which however may originate from independent analysis. Thus, drawing robust conclusions is difficult. This is further complicated by the use of different methods to classify the developmental stage of the parasite, which is sometimes reported as ‘hours post invasion’ and sometimes done by morphology (e.g., ring stage, trophozoite, schizont). In addition, when using transcriptional profiles of mRNAs as a proxy for total genome output, it is imperative to bear in mind that mRNAs may only represent a very small fraction of the total RNA. In other eukaryotic organisms, ribosomal RNAs account for approximately 80% of the total RNA content. Another approximately 15% are transfer RNAs and the remaining 5% are other RNA species, including mRNAs, non-coding RNAs, and micro RNAs (Westermann et al., 2012). But to our knowledge it is unclear how much the different RNA species contribute to the total RNA content of *Plasmodium* blood stages.

A landmark study that used microarrays to profile the *P. falciparum* transcriptome reported iconic transcriptional cascades (Bozdech et al., 2003). These cascades are thought to ensure gene expression only at times when the gene product is required. Several studies support these results, including a recent study, which profiled nascent and stabilized mRNA separately. This work reported active transcription throughout the proliferative cycle and showed that both nascent mRNA transcription and mRNA stabilization contribute to the observed transcriptional cascades (Painter et al., 2018). Moreover, several distinct periods of transcriptional bursts were identified. This ‘just-in-time’ transcription could maintain expression homeostasis, e.g., through variable promoter strength. In such a scenario, relatively strong promoters would drive expression of proteins that are needed before the DNA content increases and comparatively weak promoters would drive the expression of proteins needed after the DNA content increased. ‘Just-in-time’ transcription requires sophisticated regulation, which can be achieved by transcription factors. Only one family of 27 transcription factors was identified in the *P. falciparum* genome, the Apicomplexan AP2 transcription factors, which bind diverse DNA motifs that group functionally-related genes (Campbell et al., 2010). As the *P. falciparum* genome harbors over 5000 genes, this relatively small repertoire suggests that other regulatory mechanisms, e.g., post-transcriptional or translational, play an important role to ensure a ‘just-in-time’ expression. Indeed, transcripts for almost 90% of the *P. falciparum* genome can be detected in blood-stage parasites, including genes that are specific for the mosquito- and liver stages of the parasite’s life cycle (e.g. CSP, TRAP, SPECT2, STARP) (Le Roch et al., 2004; Otto et al., 2010; Painter et al., 2018; Toenhake et al., 2018; Chappell et al., 2020), supporting an important role for post-transcriptional regulation of gene expression.

However, a study that used nuclear run-on assays and nascent RNA sequencing to profile mRNA transcription reported that most protein-coding genes were transcribed during a single burst, suggesting an ‘all-at-once’ model of transcription (Lu et al., 2017). As the reported transcriptional activation occurred in a developmental stage that presumably harbors only a single copy of the genome, ‘all-at-once’ transcription represents a conceptually simple way to cope with the increasing DNA content. Indirect support for this model comes from studies that observed a genome-wide drop in nucleosome levels approximately at the time when the transcriptional burst was detected and nucleosomes reassembled in the stages that replicate DNA (Ponts et al., 2010; Bunnik et al., 2014; Batugedara and Le Roch, 2019). In contrast, another study reported highly similar nucleosome occupancy at different developmental stages (Kensche et al., 2015).

In an ‘all-at-once’ scenario, proteins need to be provided long after the transcriptional burst. This could be achieved through stabilized mRNAs and/or translational repression (Vembar et al., 2016). Indeed, Shock et al., 2007 reported that the mRNA half-life is substantially longer in polyploid parasites (Shock et al., 2007). This stabilization is likely accomplished by RNA-binding proteins, which make up approximately 4-10% of the *P. falciparum* genome and are increasingly transcribed in stages that replicate DNA (Painter et al., 2018). Translational repression plays an important role during other stages of the *Plasmodium* life cycle (Mair et al., 2006), but data on global and/or gene-specific translational control within one proliferative cycle in the blood stage of *P. falciparum* remains sparse. Translational repression was shown for the *P. falciparum* dihydrofolate reductase-thymidylate synthase, which binds the coding region of its own mRNA, thus repressing translation (Zhang and Rathod, 2002). More recently, it was reported that *P. falciparum* alba1 stabilizes up to 1193 transcripts and prevents their translation (Vembar et al., 2015). Although these reports on mRNA stabilization and translational repression are in line with an ‘all-at-once’ transcription, more data are needed to answer the current and emerging questions regarding transcription in *Plasmodium*.

Regardless of in favor of one or the other model of transcription, active mRNA transcription in polyploid stages was shown (Bozdech et al., 2003; Le Roch et al., 2004; Sims et al., 2009; Otto et al., 2010; Lu et al., 2017; Painter et al., 2018; Reid et al., 2018) and enzymatically active RNA polymerase II complex was detected in cells with multiple genomes (Rai et al., 2014). Moreover, dynamic histone acetylation and promoter opening can be observed throughout the proliferative cycle, further supporting active transcription in polyploid stages (Bártfai et al., 2010; Gupta et al., 2013; Josling et al., 2015; Kensche et al., 2015; Lu et al., 2017; Toenhake et al., 2018). Many of the corresponding gene products expressed at this stage play a role for erythrocyte invasion or are important to establish the intraerythrocytic compartment, in which the parasite resides. Proteins of the up to 30 daughter cells are likely needed at a relatively high copy number and it is tempting to speculate that an increasing DNA content could be beneficial for this subset of genes. Still, whether the dynamics of mRNA transcription are a valid surrogate for the total genomic output that includes all RNA species remains to be determined.

## Conclusion

*Plasmodium* parasites increase their cellular DNA content massively during one proliferative cycle. In other cells, such changes in the DNA content and, thus, the level of ploidy, are accompanied by an increased cell size and/or altered genome output. But how *Plasmodium* copes with the increasing DNA content remains elusive. Without experiments specifically designed to address this question, it is difficult to draw firm conclusions. The possible contribution of increasing cell size as a means to cope with the increased DNA content remains to be investigated in detail. The currently available data suggest a temporal separation of total RNA production and DNA replication as coping mechanism, but only quantification of genomic output over time (i.e., all different RNA species and protein) in relation to DNA content and cell volume can inform on this aspect of *Plasmodium* biology. The emerging single-cell approaches (Poran et al., 2017; Reid et al., 2018; Walzer et al., 2019), probe-independent RNA sequencing with a theoretically infinite dynamic range, and parallel quantification of the DNA content will be critical. Indeed, using spike-in RNA standards and different normalizations approaches (reads per transcriptome, per gene copy number, and per cell) were successful in detecting specific changes in transcript stoichiometry and abundance across polyploid species (Visger et al., 2019; Song et al., 2020). Addressing this aspect of *Plasmodium* biology and revealing how the parasite keeps genome output in homeostasis is an important step to understand the impact of increasing DNA content on *Plasmodium* evolution.

## Data Availability Statement

The raw data supporting the conclusions of this article will be made available by the authors, without undue reservation.

## Author Contributions

MM and MG conceived this study. MM and SS reviewed the literature. MM, SS, and MG wrote the manuscript. All authors contributed to the article and approved the submitted version.

## Funding

This work was funded by the Deutsche Forschungsgemeinschaft (DFG, German Research Foundation)—Project number 240245660—SFB 1129 and the Baden-Württemberg Foundation (ref: 1.16101.17) to MG, and the Fundação para a Ciência e Tecnologia (FCT, Portugal)—PD/BD/128002/2016 to MM.

## Conflict of Interest

The authors declare that the research was conducted in the absence of any commercial or financial relationships that could be construed as a potential conflict of interest.
